# Factors and predictors affecting late external dose rates and isolation period in patients after lutetium-177-labeled DOTA-Tyr3-octreotate treatment for neuroendocrine tumors

**DOI:** 10.1007/s12149-025-02044-5

**Published:** 2025-04-05

**Authors:** Naoto Wakabayashi, Shiro Watanabe, Satoshi Takeuchi, Takahiro Tsuchikawa, Yamato Munakata, Kenji Hirata, Rina Kimura, Junki Takenaka, Hiroshi Ishii, Kohsuke Kudo

**Affiliations:** 1 Department of Nuclear Medicine, Hokkaido University Hospital, Kita14, Nishi5, Kita-Ku, Sapporo, Hokkaido Japan; 2 Department of Diagnostic Imaging and Interventional Radiology, Hokkaido University Hospital, Kita 14, Nishi 5, Kita-Ku, Sapporo, Hokkaido Japan; 3 Department of Diagnostic Imaging, Graduate School of Medicine, Hokkaido University, Kita 15, Nishi 7, Kita-Ku, Sapporo, Hokkaido Japan; 4 Global Center for Biomedical Science and Engineering, Faculty of Medicine, Hokkaido University, Kita 15, Nishi 7, Kita-Ku, Sapporo, Hokkaido Japan; 5 Department of Medical Oncology, Faculty of Medicine and Graduate School of Medicine, Hokkaido University, Kita 15, Nishi 7, Kita-Ku, Sapporo, Hokkaido Japan; 6 Department of Gastroenterological Surgery II, Faculty of Medicine, Hokkaido University, Hokkaido University, Kita 15, Nishi 7, Kita-Ku, Sapporo, Hokkaido Japan; 7 Division of Medical Imaging and Technology, Hokkaido University Hospital, Kita14, Nishi5, Kita-Ku, Sapporo, Hokkaido Japan; 8 Neuroendocrine Tumor Center, Hokkaido University Hospital, Kita14, Nishi5, Kita-Ku, Sapporo, Hokkaido Japan

**Keywords:** [^177^Lu] DOTATATE, PRRT, Neuroendocrine tumor, [^111^in] pentetreotide, SPECT/CT

## Abstract

**Objective:**

In peptide receptor radionuclide therapy (PRRT) using lutetium-177-labeled DOTA-Tyr3-octreotate ([^177^Lu] DOTATATE), isolation is required until the external dose rate at 1 m (EDR-1 m) from the body surface falls below the regulatory standards of each country. While it is known that renal function influences EDR-1 m reduction within 180 min post-administration, the factors affecting EDR-1 m on the day following administration (Late EDR-1 m) remain unclear. This study aimed to identify factors influencing Late EDR-1 m after PRRT using [^177^Lu] DOTATATE for neuroendocrine tumors and to predict Late EDR-1 m using pretreatment [^111^In] pentetreotide single-photon emission computed tomography/computed tomography (SPECT/CT) data.

**Methods:**

This study analyzed 111 PRRT cycles administered to 36 patients between September 2021 and August 2024. Late EDR-1 m was set as the dependent variable, whereas total radiopharmaceutical uptake (LUTtotal), dose per body weight, creatinine clearance (CCr), and albumin–bilirubin (ALBI) score were set as the independent variables in the multiple regression analysis. LUTtotal was calculated using SPECT/CT data acquired after the patient left the radiation therapy room, defining the volume of interest (VOI) as the area with SUVmean + 2SD or higher in the skeletal muscle. The VOI volume multiplied by the SUVmean was used to define LUTtotal. In addition, using [^111^In] pentetreotide SPECT/CT data, the total radiopharmaceutical uptake (OCTtotal) was calculated in a manner similar to LUTtotal, and its correlation with LUTtotal was examined. A predictive equation for Late EDR-1 m was developed using the results of the multivariate analysis, and its performance was tested using subsequent cases between August 2024 and January 2025.

**Results:**

The median measured Late EDR-1 m was 8.0 (range, 4.0–26.0) μSv/h. LUTtotal and dose per body weight were significantly correlated with Late EDR-1 m, whereas CCr and ALBI scores were not. Based on the results of the multivariate analysis, the predictive equation using the dose per body weight, assuming a dosage of 7400 MBq and OCTtotal, achieved a root mean square error (RMSE) of 2.24 μSv/h. In subsequent test cases, the RMSE was 3.47 μSv/h.

**Conclusions:**

Late EDR-1 m is significantly correlated with LUTtotal and dose per body weight. It can be accurately predicted using [^111^In] pentetreotide SPECT/CT data.

## Introduction

Lutetium-177-labeled DOTA-Tyr3-octreotate ([^177^Lu] DOTATATE) is a therapeutic agent used in peptide receptor radionuclide therapy (PRRT) for neuroendocrine tumors (NETs) that are positive for somatostatin receptors (SSTRs). In some countries, patients must be isolated in a radiation therapy room after the administration of [^177^Lu] DOTATATE for radiation protection [1–3]. However, the introduction and maintenance of radiation therapy rooms with dedicated drainage and radiation shielding facilities entail significant costs, leading to a shortage of radiation therapy rooms. In Japan, from April 2022, a law amendment allowed the designation of general hospital rooms equipped with appropriate radiation protection as “a special measures patient room,” [4] enabling isolation after the administration of [^177^Lu] DOTATATE. Despite these efforts, the availability of such rooms remains limited. Furthermore, clinical studies, such as the NETTER-2 [5] and OCLUANDUM trials [6] have demonstrated the superiority of [^177^Lu] DOTATATE as a therapeutic agent for NETs. Consequently, the number of patients eligible for [^177^Lu] DOTATATE therapy is expected to increase in the future, further limiting room capacity.

Accurately predicting isolation periods is crucial to effectively manage bed control in these limited rooms. The isolation period affects the management of inpatient treatment plans and the feasibility of administering [^177^Lu] DOTATATE to other patients. Prolonged isolation periods may necessitate consideration of radiation protection during emergencies, potentially rendering treatment inappropriate depending on the patient’s condition. Conversely, predicting the isolation period enables patients to remain eligible for treatment.

Given the limited number of rooms, occupying them can prevent other patients from receiving the necessary treatment. The ability to predict isolation periods allows for optimized bed control and maximizes the number of patients who can receive treatment, including targeted radionuclide therapies beyond [^177^Lu] DOTATATE. This highlights the importance of accurate discharge planning, optimization of resource allocation, and enhanced individual patient management. Furthermore, it contributes to the protection of the public from radiation [7].

Renal function affects the external dose rate (EDR) within a short period of approximately 180 min after administration [8]. [^177^Lu] DOTATATE is primarily excreted through the kidneys, with > 60% excreted in the urine within 24 h [9], leaving almost no residue in the blood. Therefore, in patients with normal renal function, the EDR on the day after administration is expected to be primarily influenced by the amount of radionuclide uptake by the tumor. The predictability of EDR from planar images of [^111^In] pentetreotide has been evaluated [10]. However, the factors influencing EDR in the late phase, namely the day following administration, remain unknown.

This study aimed to identify factors that predict prolonged retention of radioisotopes (RIs) in patients undergoing PRRT and to identify cases where long-term radiation management is necessary. In addition, we examined whether the EDR can be predicted using [^111^In] pentetreotide single-photon emission computed tomography/computed tomography (SPECT/CT) based on the results.

## Methods

This retrospective study was conducted in accordance with the World Medical Association Declaration of Helsinki and approved by the Human Research Ethics Committee of the Hokkaido University Graduate School of Medicine and the Institutional Review Board of Hokkaido University Hospital (#022–0329). The requirement for written informed consent was waived because of the retrospective nature of this study.

The participants were 37 consecutive patients who underwent PRRT with [^177^Lu] DOTATATE for SSTR-positive NETs at Hokkaido University Hospital between October 2021 and August 2024. Data from these patients were used for the main analysis and served as training data for the EDR prediction formula. We tested the accuracy of the prediction formula using test data from nine patients who underwent their first treatment between August 2024 and January 2025.

### Peptide receptor radionuclide therapy protocol and blood sampling

Blood tests were performed within 24 h prior to treatment. The patients’ creatinine clearance (CCr) was calculated using the Cockcroft–Gault formula [11], and the albumin–bilirubin (ALBI) score was used as an indicator of liver function [12]. Granisetron was administered as an antiemetic before treatment. Renal protection was ensured by intravenous infusion of an amino acid solution containing 2.5% arginine and 2.5% lysine for 4 h [13], following granisetron administration. Approximately, 30 min after the initiation of the amino acid solution infusion, 7400 MBq of [^177^Lu] DOTATATE was intravenously administered > 30 min.

### External dose rate measurement

The administration of [^177^Lu] DOTATATE was generally scheduled between 11 AM and 2 PM on the calibration date. The following morning, the patients’ EDR was measured. External dose rate at 1 m (EDR-1 m) is defined as the 1-cm dose equivalent rate at a distance of 1 m from the body surface. EDR-1 m was measured using a calibrated ionization chamber (LUCREST ICS-1323, ALOKA, Japan) at a distance of 1 m from the abdominal surface. According to Japanese regulations, patients are informed in advance that they must remain in the radiation therapy room or the special measures patient room until the EDR-1 m becomes ≤ 18 μSv/h. The elapsed time from [^177^Lu] DOTATATE administration to measurement was approximately 20 h (19.9 ± 1.3 h). In cases where the following day was not a business day, the EDR-1 m was not measured the next morning.

### Image analysis

[^177^Lu] DOTATATE SPECT/CT images acquired on the same day after release in the radiation therapy room and [^111^In] pentetreotide SPECT/CT images taken within 6 months of the initial treatment were used for image analysis. [^111^In] pentetreotide SPECT/CT images taken 24 h after injection were used for analysis. Imaging was performed using a SPECT/CT system (Symbia Intevo Bold; Siemens Healthineers, Erlangen, Germany). SPECT scans were performed using medium-energy low-penetration collimators, photo-peak at 208 keV (187.2–228.8 keV), 60 views (30 per detector) in step-and-shoot mode with auto-contouring and 128 × 128 matrix (zoom = 1) with 2.36 mm pixel size. CT was performed using a voltage of 130 kV, quality reference of 80 mAs, pitch of 1.2, rotation time of 0.6 s and for attenuation correction reconstruction with B08 filter, and 3.0 mm slice thickness. The SPECT iterative reconstruction protocol was built using a Siemens ordered subset conjugated gradient with 24 iterations and one subset with scatter correction using the triple energy window technique. In 11 patients, imaging was performed from the abdomen to the pelvis, with no lesions identified cranial to the diaphragm, as confirmed by contrast-enhanced CT, [^18^F] fluorodeoxyglucose-positron emission tomography (FDG-PET), and [^111^In] pentetreotide SPECT/CT. In 26 patients, imaging was extended from the neck to the pelvis, with three patients presenting with lesions cranial to the diaphragm.

**Fig. 1:**
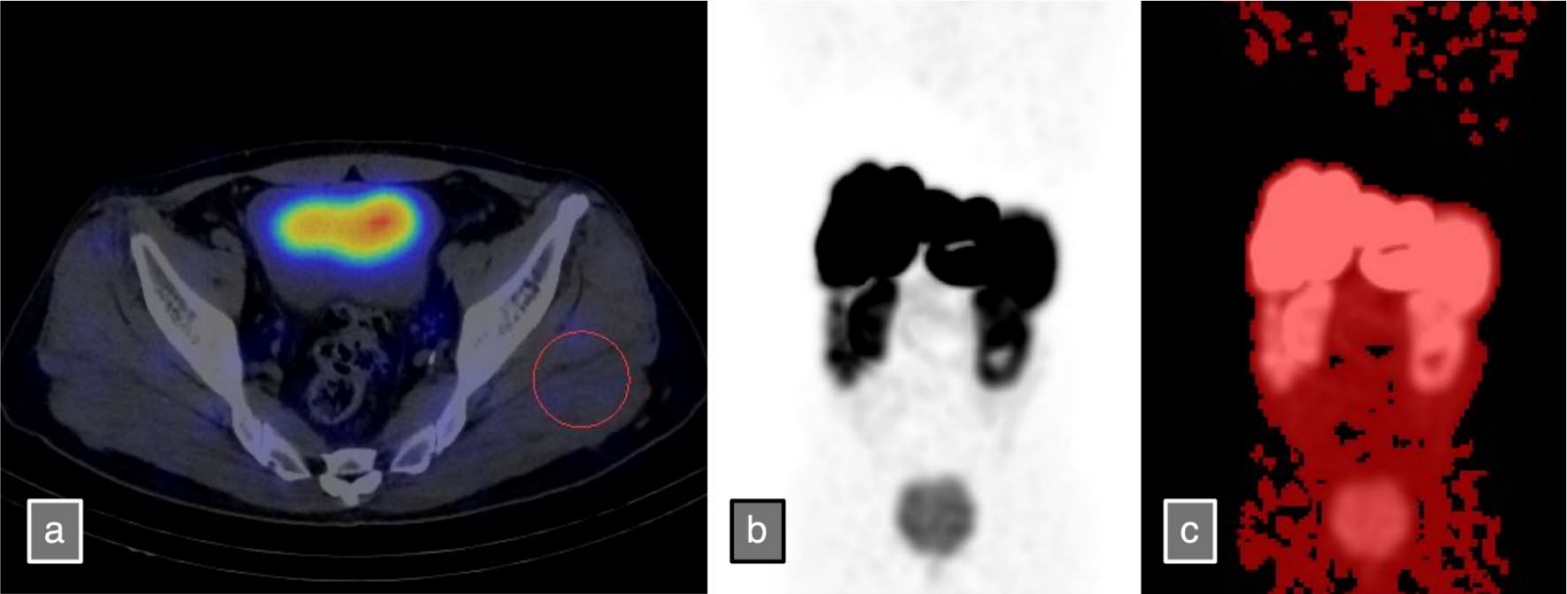
a. A case of a male in his 50 s with a pancreatic primary neuroendocrine tumor and multiple liver metastases. A [^177^Lu] DOTATATE SPECT/CT fusion image is presented. Spherical VOIs with a diameter of up to 5 cm were placed on the skeletal muscles of the gluteal region (gluteus maximus and gluteus medius), and the SUVmean and standard deviation were measured. b. [^177^Lu] DOTATATE SPECT MIP image. c. Using the open-source software Metavol, all regions with SUVmean + 2SD or higher of the skeletal muscles were set as VOIs, and LUTtotal was measured. The measured SUVmean was 0.088 with an SD of 0.059, resulting in a threshold of SUVmean + 2SD at 0.206. The SUVmean of the entire region exceeding this threshold was 3.60, and the LUTtotal was calculated as 31,062 by multiplying this value by the VOI volume. Since the total amount of RI in the body is considered to influence the external dose rate, the VOI includes not only tumor uptake but also physiological uptake in the kidneys and spleen, as well as urinary accumulation in the urinary tract. SPECT/CT, single-photon emission computed tomography; VOI, volume of interest; SUV, standardized uptake value; MIP, maximum intensity projection; LUTtotal, defined as the product of the VOI volume and its SUVmean

Image analysis was performed using the open-source software Metavol [14]. Using the SPECT/CT images, a sphere with a maximum diameter of 5 cm was placed within the gluteus maximus and gluteus medius, and the mean and standard deviation (SD) of the standardized uptake value (SUV) were calculated. The threshold was set as the mean plus 2 SD. For highly reproducible measurements, it is desirable to set the largest possible spherical volume of interest (VOI). Among the skeletal muscle regions within the imaging field, the gluteal region was selected because it allowed for the largest spherical volume to be set. While some cases could not accommodate a 5-cm sphere owing to body size, the largest possible sphere was set as appropriate. Although the gluteal skeletal muscle is close to the typical injection site for Lanreotide Acetate, no cases exhibited abnormal uptake or effects owing to calcification. For both [^177^Lu] DOTATATE and [^111^In] pentetreotide SPECT/CT, the accumulation volume within the imaging range exceeding the threshold was designated as LUTvol and OCTvol, respectively. The sum of all SUVs was designated as LUTtotal and OCTtotal, respectively, serving as the accumulation indices for the radionuclides (Fig. 1). In other words, both LUTtotal and OCTtotal correspond to the total lesion glycolysis of FDG-PET [15], using the mean background accumulation plus 2SD as the threshold.

### Data analysis

The statistical analysis software used was JMP® Pro version 17.0.0 software (SAS Institute Inc., Cary, NC, USA, 1989–2024). The significance level was set at *p* < 0.05. Multiple regression analysis was performed using the least-squares method. We defined the EDR-1 m on the day following administration as “Late EDR-1 m.” The relationship between each parameter and Late EDR-1 m was examined. The following indices were used as predictive parameters for Late EDR-1 m. CCr and ALBI scores were used as indices of drug metabolism and excretion. LUTtotal and dose per unit body weight were used as indices of [^177^Lu] uptake in the body. We created a predictive equation from the model and optimized the parameters that were indicated as independent predictors in the multivariate regression analysis and the correlation of LUTtotal and OCTtotal. We then calculated the Late EDR-1 m for 7400 MBq doses in all patients using the formula.

## Results

Patient characteristics are presented in Table 1. This study included 36 patients (17 men and 19 women). One patient who received a single treatment was excluded from the analysis because the external radiation dose rate could not be measured approximately 20 h after administration on the following day, as she left the radiotherapy room on the day of administration. All patients received standard pharmacotherapy before [^177^Lu] DOTATATE therapy, and none received [^177^Lu] DOTATATE as their initial treatment. The diseases diagnosed were gastroenteropancreatic NETs in 32 patients, pulmonary NET in 1 patient, retroperitoneal NET in 1 patient, pheochromocytoma in 1 patient, and bladder paraganglioma in 1 patient. In total, 116 administrations of [^177^Lu] DOTATATE were conducted during the study period; however, only 111 administrations were included in the analysis. The reasons for exclusion were as follows: three administrations were excluded because Late EDR-1 m was not measured the day after administration owing to non-working days, one administration was excluded owing to the unavailability of raw SPECT/CT data, and one administration was excluded because the patient left the radiation therapy room on the day of administration.

**Table 1:** Patient characteristics

Characteristic	Data	Percentage
Patient	36	
Male	17	47%
Female	19	53%
Diagnosis		
Gastroenteropancreatic neuroendocrine tumors	32	94%
Pulmonary neuroendocrine tumor	1	3%
Pheochromocytoma and paraganglioma	2	6%
Others (retroperitoneal neuroendocrine tumor)	1	3%
NET grade (*N* = 34)		
G1	6	18%
G2	24	71%
G3	4	12%
Krenning score		
3	6	17%
4	30	83%
Age at the first treatment		
Median (years)	61 (range, 46–78)	
Number of cycles of [^177^Lu] DOTATATE		
1	7	19%
2	3	8%
3	5	14%
4	22	61%
Primary lesion		
Pancreas	22	61%
Rectum	7	19%
Duodenum	1	3%
Stomach	1	3%
Small intestine	1	3%
Lung	1	3%
Bladder	1	3%
Adrenal gland	1	3%
Retroperitoneal	1	3%
Previous treatment before [^177^Lu] DOTATATE		
Surgery	20	56%
Somatostatin analogs	24	67%
Everolimus	25	69%
Sunitinib	13	36%
Streptozocin	11	31%
Transcutaneous arterial chemoembolization	1	3%
[^131^I] metaiodobenzylguanidine	2	6%
Other chemotherapy	2	6%
Target lesion		
Primary lesion	16	44%
Lymph node	9	25%
Lung metastasis	1	3%
Liver metastasis	34	94%
Bone metastasis	5	14%
Peritoneal metastasis	4	11%

The administered dose of [^177^Lu] DOTATATE was 7.32 ± 0.10 GBq, and the average elapsed time from administration to measurement was 19.9 ± 1.3 h. The Late EDR-1 m was 10.2 ± 4.9 μSv/h, with 7 administrations > 18 μSv/h. The CCr was 73.9 ± 25.5 (range, 31.0 − 146.9) mL/min. The ALBI score was –2.7 ± 0.3. The average SUVmean of the VOI for LUTtotal measurement was 1.62 ± 1.16, and the calculated LUTtotal was 15,732 ± 11,045 (range: 3,189 − 53,775).

### Factors correlating with Late EDR-1 m

**Fig. 2:**
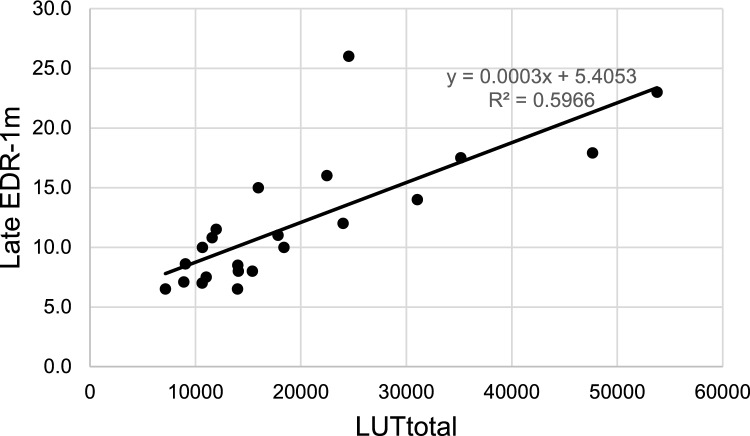
Relationship between Late EDR-1 m and LUTtotal (*N* = 111)

The results of the multiple regression analysis with LUTtotal, dose per body weight, CCr, and ALBI score as variables for Late EDR-1 are shown in Table 2. Significant correlations were observed between LUTtotal and dose per body weight. The standardized β coefficients were 0.92 for LUTtotal and 0.43 for dose per body weight. The adjusted R^2^ value was 0.80. The relationship between LUTtotal and the measured Late EDR-1 m is shown in Fig. 2.

**Table 2:** Results of multiple regression analysis (*N* = 111)

	Standardized coefficient (β) (95% CI)	t value	*p*-value	VIF
*LUTtotal	0.92 (0.81 − 1.03)	16.9	< 0.0001	1.66
*Dose per BW	0.43 (0.32 − 0.53)	8.0	< 0.0001	1.58
CCr	−0.02 (−0.13 − 0.08)	–0.47	0.64	1.50
ALBI score	0.02 (–0.08 − 0.12)	0.36	0.72	1.38

Dose per BW, dose per body weight; CCr, creatinine clearance; VIF, variance inflation factor

### Prediction of Late EDR-1 m with significant parameters

From these results, we established the following equation to calculate the remission rate from the regression: (1)
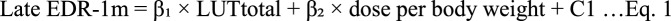


Predictive equation for LUTtotal using total octreotide accumulation: (2)
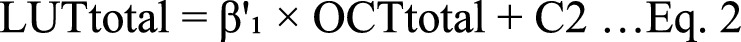


Substituting Eq. 2 into Eq. 1: (3)
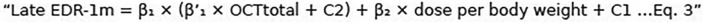


### Obtaining Eq. 3a to predict Late EDR using OCTtotal and body weight

By substituting a dose of 7400 MBq into the dose per body weight in Eq. 3, we obtained a predictive equation using two variables (OCTtotal and body weight) for the Late EDR-1 m: (3a)
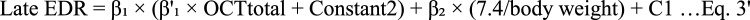


β represents the coefficient of each parameter in the multiple regression analysis. C represents the constant.

**Fig. 3:**
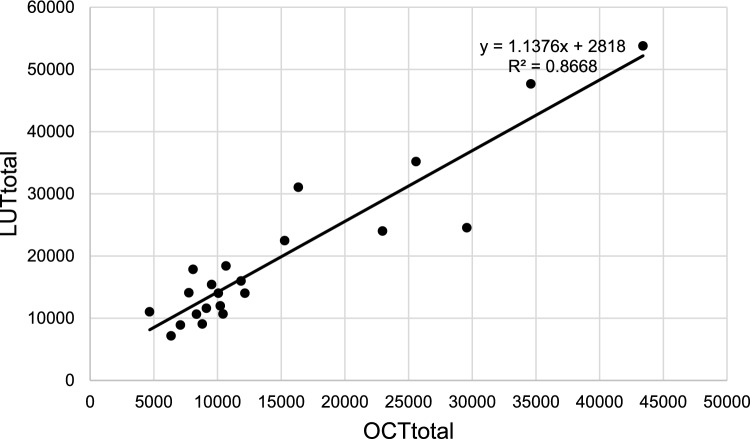
Relationship between LUTtotal and OCTtotal (*N* = 22)

**Fig. 4:**
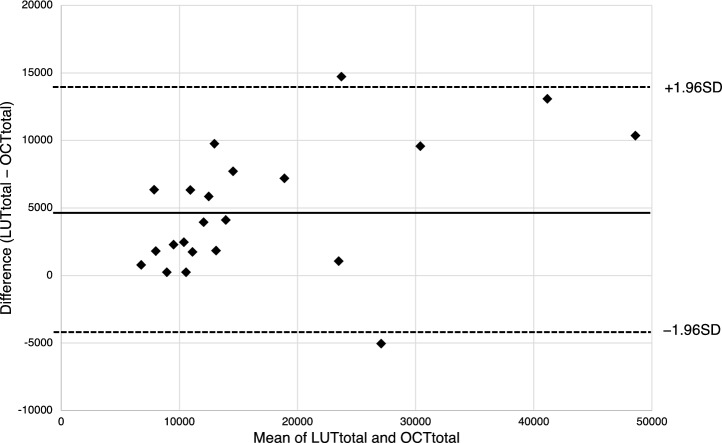
Bland–Altman plot of LUTtotal and OCTtotal (*N* = 22). The time interval between OCTtotal and LUTtotal was 62.4 ± 35.1 days. LUTtotal was imaged after the patient exited the radiation therapy room, while OCTtotal was analyzed using images acquired 24 h after injection

**Fig. 5:**
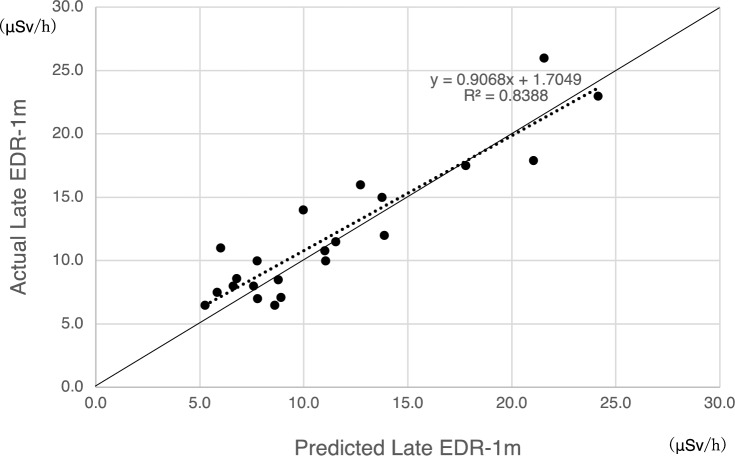
Prediction of Late EDR-1 m (*N* = 22). The relationship between OCTtotal and LUTtotal at the time of initial treatment is shown for 22 patients. The solid line represents y = x, and the dashed line represents the regression line. The adjusted R^2^ of the prediction model was 0.84, and the root mean square error was 2.24 μSv/h

**Fig. 6:**
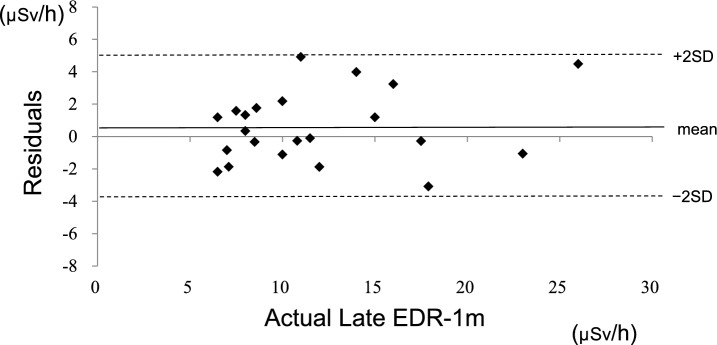
Residual plot of the prediction of Late EDR-1 m (*N* = 22)

When calculating OCTtotal, information from [^111^In] pentetreotide SPECT/CT was available for 22 patients. The SPECT/CT imaging was performed 62 ± 35 days prior to treatment. The relationship between LUTtotal and OCTtotal is demonstrated in Fig. 3, a Bland–Altman plot comparing LUTtotal and OCTtotal is demonstrated in Fig. 4, the actual values and the predicted formula for Late EDR-1 m are demonstrated in Fig. 5, and the residual plot is demonstrated in Fig. 6. The adjusted R^2^ of the prediction model from training data was 0.84, the p-value was < 0.0001, the root mean square error (RMSE) was 2.24 μSv/h, and the mean absolute error (MAE) was 1.78 μSv/h.

### Accuracy of the prediction

Among the 9 patients of test data, [^111^In] pentetreotide data were available for 7 patients. For these 7 administrations, the predicted Late EDR-1 m was calculated using the prediction formula based on OCTtotal and dose per body weight. The results are shown in Tables 3 and 4. Comparing the training data and test data, Late EDR-1 m, dose, and ALBI score were significantly lower in the test data. There were no significant differences in other parameters. The RMSE and the MAE were 3.47 μSv/h and 2.78 μSv/h, respectively.

**Table 3:** Characteristics of training data and test data for the Late EDR-1 m prediction

	Training data (*n* = 22)	Test data (*n* = 7)	*p*-value
Age	61.4 ± 8.6	53.9 ± 15.9	0.23
Body weight (kg)	61.4 ± 14.6	60.2 ± 18.7	0.91
*Dose (GBq)	7.35 ± 0.09	7.23 ± 0.05	0.001
Dose per body weight (MBq/kg)	126 ± 29	131 ± 42	1.00
*Late EDR-1 m (μSv/h)	12.7 ± 5.9	6.0 ± 1.3	< 0.001
OCTtotal	8306 ± 9871	3558 ± 2940	0.70
CCr (ml/min)	72.6 ± 25.3	93.4 ± 49.9	0.30
*ALBI Score	−2.6 ± 0.3	−3.0 ± 0.3	0.009

The values represent mean ± standard deviation

**Table 4:** Prediction of Late EDR-1 m using test data

Case	Body weight (kg)	OCTtotal	Dose per body weight (MBq/kg)	Late EDR-1 m (μSv/h)	Predicted Late EDR-1 m (μSv/h)	Absolute error (μSv/h)
1	37.4	6186	193	7.0	13.1	6.1
2	86.4	7932	83	4.5	5.3	0.8
3	66.0	9632	110	5.5	8.2	2.7
4	57.4	4486	127	5.4	7.0	1.6
5	80.5	11,530	89	8.4	7.6	0.8
6	52.9	4164	136	6.0	7.7	1.7
7	40.5	4091	177	5.2	10.9	5.8

## Discussion

This study showed that LUTtotal and dose per body weight were significant factors influencing EDR-1 m on the day after [^177^Lu] DOTATATE administration, whereas renal and liver functions (CCr and ALBI score) were not significant. EDR-1 m reflects the residual amount of RI in the body, which can be considered the sum of tumor uptake and physiological accumulation, including RI in the blood or excretory pathway. [^177^Lu] DOTATATE, intravenously administered, circulates in the bloodstream and accumulates in tumors; however, [^177^Lu] DOTATATE that does not accumulate in tumors is primarily excreted in the urine by the kidneys. LUTtotal is an indicator of accumulation in regions where the uptake is greater than the background, mainly reflecting tumor accumulation and physiological accumulation in organs, such as the spleen, kidneys, liver, and urine in the bladder.

In patients with normal renal function or moderate renal failure, which are the treatment criteria for [^177^Lu] DOTATATE, the RI in the blood and urine becomes approximately zero 24 h after administration [16]. Therefore, it is expected that Late EDR-1 m is mainly influenced by tumor uptake and physiological accumulation, and it is reasonable that LUTtotal was the most significant factor. However, most of the RIs circulating in the bloodstream that do not accumulate in tumors are excreted in the urine by the day following administration. Therefore, variations in RI excretion rates owing to renal function may influence the amount of residual RI in the body, potentially affecting the rate of decline in EDR-1 m. However, in cases of moderate renal impairment, despite possible differences in excretion rates, sufficient RI was likely excreted in the urine by the day after administration. This could explain the lack of a significant impact of renal function on the outcomes in this study. Previous reports measuring external dose rates immediately after Lu-177 administration identified sex and BMI as significant factors [17]. Notably, at the time of administration, the redistribution of the RI is not yet complete, and background accumulation remains high. Under these conditions, body shape factors influenced by sex and BMI are likely to affect external dose rates. However, in our study, approximately 20 h had elapsed after administration, during which background accumulation had significantly decreased. Therefore, the influence of body composition is considered limited.

In studies on [^131^I] therapy for thyroid cancer, patients who have undergone ablation (those with residual thyroid tissue, including the thyroid bed) exhibit a biphasic decrease in the EDR, whereas follow-up patients (those receiving their second or subsequent treatments) exhibit a monophasic decrease [18]. The biphasic decrease is believed to be due to the rapid redistribution of RI from the stomach to the thyroid, causing the RI to shift away from the excretory pathways of the blood, urine, and intestines. In case of a monophasic decrease, the authors suggested that the rate of EDR reduction was dependent on excretion via the kidneys and intestines. One of the key differences between [^131^I] therapy and [^177^Lu] DOTATATE therapy is that the latter targets patients with lesions that show high uptake. [^177^Lu] DOTATATE demonstrates more specific accumulation in tumors than [^131^I] sodium iodide, and it is believed that unbound [^177^Lu] DOTATATE in the bloodstream is rapidly excreted through the kidneys. Therefore, the decline in EDR during [^177^Lu] DOTATATE therapy is expected to be biphasic, as reported by Levart et al. [19]. Based on these observations, the first phase of excretion corresponds to the period shortly after administration, approximately within the same day, and the rate of excretion is dependent on renal function. The second phase, or accumulation phase, occurs after renal excretion has mostly ceased, and the RI concentration in the blood drops to significantly low levels. The EDR in this phase reflects the amount of RI accumulated in the body, primarily in the tumors.

OCTtotal was significantly correlated with LUTtotal. Both [^111^In] pentetreotide and [^177^Lu] DOTATATE are ligands with an affinity for SSTR and are conjugated with RIs that emit gamma rays, which can be used for SSTR imaging via SPECT. Although there are differences in the affinity for each SSTR subtype, both have a strong affinity for SSTR2 [20, 21], which is frequently expressed in many NETs. Therefore, we believe that the differences in affinity for SSTRs, including subtypes other than SSTR-2, were limited in this study. Therefore, it was possible to predict the Late EDR-1 m by substituting the relationship between LUTtotal and OCTtotal into the regression equation derived from LUTtotal.

Takai et al. previously reported that it is possible to predict patients who may not meet the EDR-1 m standards in radiation therapy rooms on the day after administration, based on planar imaging using [^111^In] pentetreotide [10]. They converted the pixel values of planar images into radioactivity; if the sum of the calculated radioactivity exceeded a certain threshold, the EDR was likely to surpass the discharge criteria. Although EDR reflects the amount of RI accumulated in the body, it is hypothesized that the 24 h post-administration planar image of [^111^In] pentetreotide approximates the planar image taken on the day following [^177^Lu] DOTATATE administration, thus enabling prediction. In this study, we investigated the factors that contributed to high RI accumulation on the day following administration. LUTtotal was the most significant factor, whereas renal function was not a significant factor. Furthermore, we demonstrated that it is possible to equal or greater accurately predict EDR-1 m levels on the day after administration using [^111^In] pentetreotide SPECT/CT and the patient’s body weight. These findings are consistent with those of previous studies and represent a reasonable outcome. However, the predictive performance was not satisfactory for the test data. Compared to the training data, the test data showed significantly lower Late EDR-1 m, dose, ALBI score, and OCTtotal, suggesting that the sample had a tendency toward a lower tumor burden. In addition, the cases with particularly large errors were all in patients with low body weight, indicating that Late EDR-1 m was overestimated in this population. This is likely due to a bias in the training data, as patients with lower body weight tended to have a high tumor burden, resulting in elevated Late EDR-1 m. It is expected that as the number of cases increases, this bias will be mitigated, leading to improved predictive performance. Furthermore, we found that LUTtotal and EDR showed a good correlation. LUTtotal primarily indicates the accumulation of RI in the body, which is thought to reflect the amount of viable tumor tissue. Therefore, tracking changes in EDR-1 m over multiple administrations may provide a simple method for assessing changes in the amount of viable tumor tissue, which can be useful for predicting treatment efficacy and prognosis without SPECT/CT.

### Limitations

This was a single-center retrospective study with a small sample size. The dataset includes multiple treatments for the same patient, with varying treatment numbers for each patient, resulting in a possibility of bias in the sample. We used the SUV from SPECT, the quantification of which has been a subject of debate; however, we made efforts to minimize this effect by setting a threshold based on skeletal muscle uptake. This analysis utilized SPECT/CT data from a single institution using the same imaging equipment. Therefore, the applicability of the study results to data from other institutions or different imaging devices may be limited. Furthermore, the time elapsed between administration and measurement was not included in the analysis. As EDR-1 m reflects the amount of RI in the body, it is evident that the longer the elapsed time, the more the RI decreases owing to radioactive decay or tumor shrinkage, which can influence EDR-1 m. However, the measurements were obtained approximately 20 h after administration; therefore, we did not include this variable. This study retrospectively analyzed data on EDR measurements related to patient discharge from the radiation therapy room. Due to hospital ward logistics, staff availability, and patient management considerations, the measurements were conducted approximately 20 h after administration. However, information on EDR at earlier or later time points is not available. For future outpatient treatments, additional data at different time points are required to improve dose rate predictions. For a more accurate analysis, it is desirable to increase the number of cases and conduct a study using [^68^ Ga] DOTATATE PET, including the elapsed time to measurement.

## Conclusion

In the treatment of NETs with [^177^Lu] DOTATATE, the primary factor influencing EDR-1 m on the day after administration is the amount of RI uptake in the body. The renal function (CCr) and liver function (ALBI) are not significant factors. These findings suggest the potential of predicting the EDR on the day after administration using information from [^111^In] pentetreotide.

## Data Availability

All data used to support the findings of this study are included within the article.
